# Contraception awareness and practice among antenatal attendees in Uyo, Nigeria

**Published:** 2011-12-06

**Authors:** Augustine Vincent Umoh, Mathias Gabriel Abah

**Affiliations:** 1 Department of Obstetrics and Gynaecology, University of Uyo Teaching Hospital, Uyo, Nigeria

**Keywords:** Contraception, prevalence, awareness, antenatal care, maternal mortality, Nigeria

## Abstract

**Introduction:**

Contraception is major component of reproductive health. The study aims to document the awareness of contraception and its use in Uyo, South-south Nigeria and provide useful information for future intervention strategies.

**Methods:**

A cross-sectional study using pretested questionnaires among antenatal attendees in a tertiary and a secondary health facility in Uyo.

**Results:**

A total of 550 women took part in the study. Majority of respondents (92.4%) were aware of contraception while 52.6% had ever used any form of contraception. The condom (60.3%) and the pill (49.9%) were the most common forms of contraception that the women had heard of, mostly from the doctor (36.9%), radio (33.8%) and nurse (28.5%). The condom (46.7%), withdrawal method (14.1%) and the pills (13.3%) were the most commonly used forms of contraception. Majority of the women (70.5%) planned to use contraception in the future and this intention was significantly related to the woman's educational status (p<0.05) but not to religion or occupation. Fear of side effects, uncertainty about its need, partner objection and previous side effects were the common reasons given for unwillingness to use contraception in the future.

**Conclusion:**

Our study has shown that while there is good contraceptive awareness in Uyo, Nigeria, this is not matched by commensurate contraceptive prevalence but prospects for improvement exist. There's need to tackle known obstacles to contraceptive uptake. Also targeted campaigns and every available opportunity should be used to provide reproductive counseling to women especially on contraception.

## Introduction

Maternal mortality and morbidity have been noted to be prevalent in sub-Saharan Africa and other resource-poor and underdeveloped nations of the world [1]. Unplanned pregnancy and unsafe abortion are major contributors to these dismal health indices and are themselves direct consequences of failure or non use of contraception [1,2]. Unsafe abortion accounts for about 11% of maternal mortality worldwide and up to 40% in Nigeria [1,2], where contraceptive prevalence has been reported to be very low [3–5].

It is well known that contraceptive practice can improve maternal health and it has continued to recur in most strategies proposed or developed by world bodies to achieve improved maternal health worldwide [6,7]. Family planning, which can be achieved through contraception, is one of the pillars of the Safe Motherhood Initiatives (SMI) introduced in 1987 to reduce maternal morbidity and mortality while contraceptive prevalence rate is one of the indices for assessing the achievement of universal access to reproductive health in the Millennium Development Goals [6,7].

Generally, it has been observed that contraceptive awareness or knowledge is high among Nigerian women of all age groups studied [4,5,8]. Method awareness however varies with the studied population with most people knowing more about condoms, oral contraceptives and the intrauterine contraceptive device; their sources of information being friends, books/ magazines, radio and relatives [9].

Unfortunately, despite a good contraceptive awareness, studies have shown a disappointingly very low contraceptive usage in Nigeria [3–5]. This is believed to be due to several barriers including lack of access, socio-cultural and religious factors, partner‘s opposition and fear of side effects of contraceptives [10].

This study was carried out among antenatal clinic attendees in the two main public hospitals providing obstetric care at secondary and tertiary levels in the urban city of Uyo metropolis. The choice of the centers we believe will give a broader perspective of the real situation in Uyo, South-south Nigeria as a result of the mixed socio-demographic characteristics of the clients from these institutions. We studied the general and method specific contraceptive awareness, the sources of their knowledge as well as both the general and method specific contraceptive prevalence. Information obtained from the study will help to enrich the knowledge base on contraception in our environment as well as sharpen the content of antenatal health education and other interventions.

## Methods

### Setting

Uyo is the capital city of Akwa Ibom State in the Niger Delta region in the South-south geopolitical region of Nigeria. The people are mainly Christians, monogamous and of the Ibibio/Efik speaking stock. The state has a population of about 4 million people and is served by one tertiary health institution, the University of Uyo teaching hospital (UUTH). Another health institution, the St Lukes Hospital, Anua, Uyo functions as a secondary health facility in the capital city and enjoys good patronage from women of the middle and lower socio economic class as the cost of services, especially for maternity services, for which it is reputed for, is much cheaper. This study was approved by The University of Uyo Teaching Hospital Institutional Review Board.

### Sources of data and Analysis

This was a cross sectional questionnaire-based survey. Data was obtained from women attending antenatal care in both the University of Uyo Teaching Hospital and St Luke's hospital, Anua. A questionnaire instrument which was pre-tested among antenatal clinic attendees and nurses in the maternity unit of the Teaching hospital was used for data collection. The questionnaires were self-administered but a set of nurses in the clinics of both hospitals were trained to offer assistance to those who may require them especially those who may not be literate. The questionnaires were offered to all women attending the clinic during the study period after verbal consent was sought and obtained. The only exclusion criterion was denial of consent. The questionnaires covered areas of socioeconomic characteristics of respondents, their fertility profiles and intentions as well as contraceptive awareness, knowledge and practice. Data obtained were analysed using the SPSS 17 statistical package for Windows. Level of significance was set at p

## Results

A total of 550 questionnaires were administered out of which 522 were correctly filled and were suitable, and used, for analysis – 330 from UUTH and 192 from Anua.

### Socio-demographic characteristics

Majority of clients (90.8%) were between the ages of 20 – 34 years with a mean age of 27.75 years. Also majority of respondents (67.7%) were of the predominant Ibibio tribe, while the Anang and Igbo tribes constituted 11.4% and 10.3% respectively. The married respondents were in the majority (93.7%) while those engaged constituted 3.3% and the single ones were 1.7%. Out of those married respondents, 96.9% were in their first marriage and 97.5% were in monogamous relationships. However, 25.6% of the women were born into polygamous homes. The Pentecostal was the most prevalent religious affiliation among respondents (53.3%). Other religious affiliations were Catholic 18.9%, spiritual 10.5%, protestant 8.9% and Islam 1.4%. Students constituted 28.6% of respondents while civil/public servants, sales/trading, professional and fulltime housewives were 24.1%, 17%, 4.7% and 4.9% respectively. Those unemployed were 11.4%. About 90% of respondents had at least a secondary level education out of which 44.3% had had a university education.

### Contraception awareness and usage

Most of the women (92.4%) were aware of contraception while 52.6% had ever used a form of contraception. The condom (60.3%), pill (49.9%), injectables (38.2%), withdrawal method (34.9%) and intra-uterine contraceptive device IUCD (28.7%) were the most common forms of contraception that women had heard of. Others are outlined in Table 1.

**Table 1:** Methods of contraception that antenatal in Uyo women have heard of and ever used

Types of contraception	Awareness (%)	Usage (%)
Condom	60.3	46.7
Pills	49.9	13.2
Injectables	38.2	7.9
Withdrawal	34.9	14.1
IUCD	28.7	6.6
Female condom	22.3	0.0
Breastfeeding	20.3	2.9
Diaphragm	19.4	0.0
Periodic abstinence	19.2	9.5
Total abstinence	16.3	0.0
Implants	14.3	0.0
Rhythm/Natural method	13.7	4.5
Male Sterilization	11.5	Not applicable
Female sterilization	11.3	0.0
Traditional methods	9.5	1.2
Foam/jelly	9.3	0.0

Majority of the women got to hear about these methods of contraception form their doctors (36.9%), while others heard it from the radio (33.8%), nurse (28.5%), friends (21.2%) newspapers (18.8%) and the television (17.9%; Table 2).

**Table 2:** Sources of contraceptive information among antenatal women in Uyo

Source	Percentage
Doctor	36.9
Radio	33.8
Nurse	28.5
Friend	21.2
Newspapers	18.8
TV	17.9
Teachers	9.7
Relation	5.2
Mother	5.3
Neighbour	4.4
Classmate	4.2
Chemist	2.9
Parents	2.6
Pastor	1.1
Father	0.9
Siblings	0.4

The most common form of contraception that the women had ever used was the condom (46.7%). Others were the withdrawal method (14.1%), pills (13.2%), periodic abstinence (9.5%), injections (7.9%) and IUCD (6.6%; Table 1). None of the women had ever used the implant, diaphragm, female condom and foam/jelly.

### Future intentions to use contraception

Majority of women (70.5%) planned to use some form of contraception in the future to plan their family. The intention to use contraception in the future was significantly related to the level of education (p0.5) or occupation (p>0.5). The more educated the respondent the greater the greater the likelihood to report an intention to use contraception in the future. Among the 29.5% who do not intend to use contraception in the future, the most common reason given was the fear of side effects (39.3%) while some others believed that they do not need contraception (20.7%). Other reasons given were objection of husband/partner (9.6%) and previous side effects (9.6%) and against religious believe (7.4%; Table 3).

**Table 3:** Reasons for not wishing to use contraception in the future among antenatal women in Uyo

Reason	Percentage
Fear of side effects	39.3
I don't think I need it	20.7
Objection of husband/partner	9.6
Previous side effects	9.6
Against religious believe	7.4
Health reasons	7.4
Don't know where to get service	5.9
Cannot afford the price	1.5
Objection by other family members	0.7
I don't expect to have sex	0.7

Other reasons given include age, was told that condom is not effective, it is accompanied by heat, if I tell God he is able to stop pregnancy, I don't understand any method and fear of use of any method.

## Discussion

We found from this study that 92.4% of our respondents have heard of contraception, family planning or some ways to delay/ avoid pregnancy. This high contraceptive awareness is in conformity with reports from other studies across Nigeria [4,5,8,9,11] irrespective of the population used but is much higher than reported national average figures of 54.7% by Oye-Adeniran et al and 72.1% by the NDHS 2008 [4,5]. It is however, similar to the findings in the south south geopolitical zone of which Uyo is a part of [4]. In the 2008 NDHS, knowledge of contraceptive methods was 89.9% among women from the south south geopolitical zone of Nigeria and 98.2% of women with post-secondary education had heard of at least a method of contraception [4]. This higher awareness may be also be due to the high educational level of the respondents in this study, majority (90.9%) of whom have at least secondary education and 44% being graduates.

Our study also showed that the methods of contraception that women have heard of most were condoms (60.3%), pills (49.9%), injectables (38.2%), and intrauterine contraceptive devices (28.7%). Other studies also revealed method specific awareness being highest for pills, condoms, injectables and IUCD [4,5,11]. The withdrawal method was considerably high (34.9%) in this study. This may be partly due to the fact that one of the sites used actively promotes ‘natural family planning’. The methods least known were the traditional methods, female and male sterilization and the implants. This is similar to findings from other studies [4,11]. The poor knowledge of the sterilization as method of contraception may not be unrelated to its low level of acceptance and practice [12] due to cost, the need for surgery to which many women in our environment are averse to and the fact that it is a permanent method amongst many other reasons. As a very effective mode of contraception it is important to raise awareness on it and also provide the enabling environment for practice considering the high total fertility rate (TFR) of 5.7 in Nigeria [4] and the implications for our socioeconomic growth.

Information about contraception was obtained from varying sources in our study mainly from doctors (36.9%), radio (33.8%), nurse (28.5%), friend (21.1%), newspaper (18.8%) and television (17.0%). These sources were similar to those reported in other studies [11,13]. However, in terms of distribution it was different from other reports where friends, radio and books/magazines were the most common sources [9,13]. This difference may be due to the difference in the study population, ours being among pregnant women who currently are having contact with health workers while others were among younger age group and women in the community. That the health workers are providing information must be commended and encouraged as the antenatal period is, and must be made, a good entry point for reproductive health information and counseling. When information is received from health workers there is a greater likelihood that appropriate information would be provided compared to those received from friends who may be as ignorant as the woman herself.

The general contraceptive (ever use) prevalence was 52.4% from our study. This provides a measure of the cumulative experience of a population with family planning which in the NDHS 2008 was 29% [4]. A very low ever use (cumulative) prevalence of 9.3% was found among secondary school girls, and 11.1% among young women in Nigeria [5,9]. This contrast is not surprising as ever use generally increases sharply with increasing age to the peak of reproduction then slowly thereafter [4.] Most studies in Nigeria have shown very low contraceptive prevalence that does not match the very high contraceptive awareness [5,9,11,14,15]. This findings are a sharp contrast to those in the United states of America where more than 99% of women will have used at least one contraceptive method at some point in time, making contraception and family planning an important aspect of preventive health care for women [16,17] Even though, quoted figures from other studies above[4,5,9] are low, 52.4% found in our study could be considered to be significantly high given as it represents the cumulative experience of our studied population on contraception. It's well known that current use is a better measure of contraceptive prevalence as it measures the actual contraceptive practice at a particular point in time and correctly determines the reduction of fertility attributable to the contraceptive. However, a prevalence study of contraception is impossible among our study population, but it is obvious that a woman who has ever used contraception is more likely to use in future. The geopolitical area (South south, Nigeria) from which this study was carried out was also noted to have very high contraceptive general and method awareness [4] just as we have found in this work.

The method of contraception most commonly used by our respondents was the condom (46.7%). This was also the finding by other workers in Nigeria [4,5,11]. Other methods used, albeit less commonly, included the withdrawal method (14%) and the pill (13%). The pill has also been found to be used frequently in other studies [4,5] and a study among female undergraduate students in Nigeria showed that 45% used withdrawal method to prevent conception with only 26% using condoms [18]. However, the use of the withdrawal method by a proportionately larger number of women in our study gives cause for concern considering its very high failure rate and the lack of protection against sexually transmitted infection including human immunodeficiency virus (HIV) for which our study area currently has the second highest prevalence in Nigeria [19].

It is encouraging to observe that many (70.5%) of our respondents intend to use a method to plan the number of children they have in future. The implication of this is that contraceptive prevalence is likely to increase in future with its attendant effect on fertility rate. This expected increase however does not obviate the need for campaigns to expand the reach and uptake of contraception to all sexually active women.

Twenty-nine percent of Uyo women from this study declared a non-readiness to use any form of contraception in future. Of the several reasons given by our respondents for not wishing to use contraception, fear of side effects (39%) and not being certain if they need it (21%) stand out. The fear of side effects has been a recurring factor reported in many other studies [10,13]. Other reasons given include problem of source, religious believe, spousal objection, previous side effects and other health reasons. These issues need to be addressed in campaigns to improve uptake of contraception. Several obstacles have been documented to affect contraceptive prevalence in less developed nations including Nigeria over the years and recommendations have been made for improvement [10,18,20]. Sadly however, these constraints have continued to persist and hamper the uptake contraception.

## Conclusion

Our study has shown that while there is good contraceptive awareness in Uyo, Nigeria, this has not been matched by commensurate contraceptive prevalence but prospects for improvement exist. The known obstacles to contraceptive uptake are still relevant and measures to overcome them may be the main determinants in improving the contraceptive prevalence rate and consequently the total fertility rate. In addition to targeted campaigns, every available opportunity should be used to provide reproductive counseling to women especially on contraception.

## References

[1] Maternal mortality in 2005 Estimates developed by WHO, UNiCEF, UNFPA and the World Bank. http://www.who.int/whosis/mme.

[2] World Health Organisation (WHO) (2004). Unsafe abortion. Global and regional estimates of the incidence of unsafe abortion and associated mortality in 2000. http://whqlibdoc.who.int/publications/2004.

[3] Oye-Adeniran BA, Adewole IF, Umoh AV, Oladokun A, Ghadegsin A, Ekanem EE, Yusuf B, Odeyemi KA, Iwere N, Mahmoud P (2006). Community-based study of contraceptive behaviour in Nigeria. Afr J Reprod Health.

[4] National Population Commission (NPC) [Nigeria] and ICF Macro (2009). Nigeria Demographic and Health Survey 2008.

[5] Oye-Adeniran BA, Adewole IF, Odeyemi KA, Ekanem EE, Umoh AV (2005). Contraceptive prevalence among young women in Nigeria. J Obstet Gynaecol.

[6] World Bank, WHO, UNFPA (1987). Preventing the Tragedy of Maternal Deaths: A Report on the International Safe Motherhood Conference.

[7] Goag 5: Countdoun to 2015 (2007). Tracking the millennium development goals. http://www.mdgmonitor.org.

[8] Abiodun OM, Balogun OR (2009). Sexual activity and contraceptive use among young female students of tertiary educational institution in Ilorin. Nigeria. Contraception.

[9] Bassey EA, Abasiattai AM, Asuquo EE, Udoma EJ, Oyo-lta A (2005). Awareness, attitudes and practice of contraception among secondary school girls in Calabar. Niger J Med.

[10] Carr D, Khan M (2004). The Unfinished Agenda: Meeting the needs for family planning in less developed countries.

[11] Oyedokun AO (2007). Determinants of Contraceptive Usage: Lessons from Women in Osun State. Nigeria J Humanities Soc Sci..

[12] Oye-Adeniran BA, Umoh AV, Ogedengbe OK, Odum CU, Nnatu SNN (2000). Female Surgical Contraception at the Lagos University Teaching Hospital, Lagos, Nigeria: A Five Year Review. Nigerian Quarterly Journal of Hospital Medicine.

[13] Oye-Adeniran BA, Adewole IF, Umoh AV, Oladokun A, Gbadegesin A, Odeyemi KA (2005). Sources of contraceptive commodities for users in Nigeria. PLoS Med..

[14] Amazigo U, Silva N, Kaufman J, Obikeze DS (1997). Sexual activity and contraceptive knowledge and use among in-school adolescents in Nigeria. Int Fam Plan Persp..

[15] Okpani AO, Okpani JU (2000). Sexual activity and contraceptive use among female adolescents: A report from Port Harcourt. Afr J Reprod Health.

[16] Mosher WD, Jones J (2010). Use of contraception in the United States: 1982–2008. National Center for Health Statistics. Vital Health Stat.

[17] Miklavcic AY (2011). Over-the-counter contraceptives. Postgrad Obstet Gynaecol..

[18] Aziken ME, Okonta PI, Ande AB (2003). Knowledge and Perception of Emergency Contraception Among Female Nigerian Undergraduates. International Family Planning Perspectives.

[19] Federal Ministry of Health, Nigeria (2010). Technical report on the 2010 National HIV sero-prevalence sentinel survey among pregnant women attending antenatal clinics in Nigeria.

[20] Ogbaje Elizabeth, Igharo Elizabeth (2009). Contraceptive Security in Nigeria: Assessing Strengths and Weaknesses.

